# Prediction of cytochrome P450 isoform responsible for metabolizing a drug molecule

**DOI:** 10.1186/1471-2210-10-8

**Published:** 2010-07-16

**Authors:** Nitish K Mishra, Sandhya Agarwal, Gajendra PS Raghava

**Affiliations:** 1 Bioinformatics Centre, Institute of Microbial Technology, Chandigarh, India

## Abstract

**Background:**

Different isoforms of Cytochrome P450 (CYP) metabolized different types of substrates (or drugs molecule) and make them soluble during biotransformation. Therefore, fate of any drug molecule depends on how they are treated or metabolized by CYP isoform. There is a need to develop models for predicting substrate specificity of major isoforms of P450, in order to understand whether a given drug will be metabolized or not. This paper describes an *in-silico *method for predicting the metabolizing capability of major isoforms (e.g. CYP 3A4, 2D6, 1A2, 2C9 and 2C19).

**Results:**

All models were trained and tested on 226 approved drug molecules. Firstly, 2392 molecular descriptors for each drug molecule were calculated using various softwares. Secondly, best 41 descriptors were selected using general and genetic algorithm. Thirdly, Support Vector Machine (SVM) based QSAR models were developed using 41 best descriptors and achieved an average accuracy of 86.02%, evaluated using fivefold cross-validation. We have also evaluated the performance of our model on an independent dataset of 146 drug molecules and achieved average accuracy 70.55%. In addition, SVM based models were developed using 26 Chemistry Development Kit (CDK) molecular descriptors and achieved an average accuracy of 86.60%.

**Conclusions:**

This study demonstrates that SVM based QSAR model can predict substrate specificity of major CYP isoforms with high accuracy. These models can be used to predict isoform responsible for metabolizing a drug molecule. Thus these models can used to understand whether a molecule will be metabolized or not. This is possible to develop highly accurate models for predicting substrate specificity of major isoforms using CDK descriptors. A web server MetaPred has been developed for predicting metabolizing isoform of a drug molecule http://crdd.osdd.net/raghava/metapred/.

## Background

Metabolism determinates the fate of a compound entering inside the body. Ideally, drugs are broken down to harmless soluble metabolites that are easily excreted through urine or bile [1]. Drug metabolism is a process, which play vital role in pharmacokinetics and therapeutic action of drug molecules [2]. Cytochrome P450 enzymes (CYPs) are a multi gene family of heme-containing isoenzymes that are involved in oxidative metabolism of drug, steroids and carcinogens. About sixty CYPs are reported in human genome, but more than 90% of all therapeutic drugs are metabolized by five isoforms i.e. CYP 3A4, 2D6, 1A2, 2C9 and 2C19 [3,4].

In the past, several methods have been developed for predicting the metabolism of drug molecules using machine learning techniques. Haji-Memonian *et al*. [5] developed a CoMFA based method for predicting substrate specificity of CYP2D6 isoform in which the models were trained on 24 substrates. Blakin et al [6] used Kohonen self-organizing maps and trained their models on 33 molecules. Crivori and Proggesi *et al*. [7] used experimental Km for developing quantitative structure metabolism relationship (QSMRs) model. Manga *et al*. [8] developed QSAR model for the determination of the P450 enzyme predominantly responsible for a drug's metabolism. Most of aforementioned approaches focused on Michaelis constant K_m _values for the prediction of isoform specificity and were developed on small number of substrates. Yap *et al*. [9] used SVM for developing model for predicting inhibitors and substrate of three isoforms (i.e CYP 3A4, 2C9 and 2D6), where Ki value was used to build SVM regression model.

There are two major problems in the existing methods; i) most of them developed on limited number of substrate/drugs and ii) developed for limited number of isoforms. Recently Terfloth *et al*. [10] developed SVM based QSAR model for predicting isoform specificity of three major CYPs i.e CYP 3A4, 2C9 and 2D6. They used 146 compounds for training their models which was developed using multinomial logistic regression, decision tree, or support vector machine (SVM). We manually examined 126 molecules out of 146 reported in DrugBank 2.5 [11,12] and found that 63 molecules were metabolized by more than one isoform.

In this study, we have investigated the isoform specificity of five major isoforms CYP 3A4, 2D6, 1A2, 2C9 and 2C19 that are responsible for metabolizing more than 90% drug molecules [3,4]. We have developed all models on a clean and large dataset which was created from the latest release of DrugBank. In the present study, we have developed two types of model - i) single label models where the model predicts best single metabolizing isoform for a drug molecule and ii) multi label models where the model predicts a number of metabolizing isoform for a drug. First time, we have developed model for two very important Cytochrome P450 isoform, CYP1A2 and CYP2C19. All the models were evaluated using cross-validation techniques and on an independent dataset. First time an attempt has been made to develop web server for predicting metabolizing isoforms for a drug molecule.

## Methods

### Main dataset

All substrates that are metabolized by any of the following isoform CYP 3A4, 2D6, 1A2, 2C9 and 2C19 were obtained from DrugBank2.5 [11,12]. We have obtained a total of 372 drug molecules where each of these molecules was metabolized by at least one of the five isoforms. In order to create an exclusive dataset, we remove all those molecules that are metabolized by more than one isoforms. Finally, we got a dataset of 216 drug molecules, which consists of 111, 47, 29, 20 and 19 molecules metabolized through CYP 3A4, 2D6, 1A2, 2C9 and 2C19 isoforms respectively.

### Independent dataset

We have created an independent dataset in order to evaluate performance without any bias. For this, we downloaded from DrugBank 146 molecules that were reported to be metabolizing by one or more isoform used in this study. This independent dataset consists of total 146 molecules, where 92, 74, 41, 47 and 49 molecules have metabolic specificity for CYP 3A4, 2D6, 1A2, 2C9 and 2C19 isoform respectively.

Names of the molecules used in main dataset and independent dataset are given in Additional file 1: Table S1 - S6.

#### Molecular Structure and Descriptor Calculation

The 2D structure of each molecule was downloaded in Mol file format from DrugBank. These 2D structures were converted into 3D structure using CORINA software [13], followed by energy minimization using Hamiltonian parameter AM1. Each minimized molecular structure was treated as energetically preferred 3D structure for QSAR study. We used TSAR-3.3 [14] for computing 1D, 2D and 3D descriptors belonging to different categories such as mass, surface area, volume, moment of inertia, dipole, molar refractivity, lipophilicity, lipoles, connectivity, electrostatic parameters. In addition, ADMEWORKS Model Builder version 3.0 [15] were also used for calculating of 2192 molecular descriptors categorized into different descriptor block such as topological descriptors, constitutional descriptors, geometrical descriptors, physiochemical and electrostatic descriptors. The main categories are charge, charged polar surface area (CPSA), CPSA-AM1, carbon type (CTYPE), molecular distance edge descriptors, electro topological status index, conformational flexibility, geometrical moments, gravitation index descriptors, kappa index, path count, molecular connectivity, molar refractivity substructure descriptors, hydrogen bond specific descriptors, HMO (Huckle molecular orbital) descriptors, MOPAC descriptors. Finally we got total 2392 molecular descriptors mainly computed using ADMEWORKS Model Builder and TSAR.

### Selection of Descriptors

One of the major challenges in a QSAR is the selection of relevant molecular descriptors from large number of descriptors. We removed descriptors; i) having more than 5% missing value, ii) contain less than 10% non-zero value, iii) high correlation (≥ 0.9 Pearson's correlation) with other descriptors and iv) multicollinearity (i.e. descriptors are pair wise correlated in a multiple regression model such as stepwise MLR or leap and bound MLR.) is more than 0.95. This way, numbers of molecular descriptors were reduced to 113. The numbers of descriptor were further reduced using genetic algorithm (GA), a powerful variable selection approach [16,17]. Finally, we obtained 41 top ranking descriptors that were used for developing SVM based QSAR models (Additional file 1: Table S7).

### Cross-validation Techniques

The performances of QSAR models were evaluated using leave one out cross-validation (LOOCV) and fivefold cross-validation techniques. In LOOCV, model is trained on N-1, where N is the total number of examples and performance is tested on the remaining examples. This process is repeated in such a way that each example is used once for testing. In five fold cross-validation, data set is randomly divided in five partitions of similar size. The training and testing were carries out 5 times, each time using one set in testing and remaining four sets for training [18,19]. The model is rebuilt five times, one for each fold ensuring that all molecules are used for testing once.

### Figure of merits

In order to assess the performance of the models, we used several parameters that include sensitivity, specificity, accuracy, and MCC. Sensitivity is the percentage coverage of correctly predicted CYP isoform substrates; specificity is the percentage coverage of predicted non-CYP isoform substrates; accuracy is the percentage of correctly predicted biotransformation of drug molecules and MCC is the fitness function for model optimization. These parameters can be represented by following equations:

$$\begin{array}{l} \text{Sensitivity}=\frac{\text{TP}}{\text{TP}+\text{FN}}\times \text{100}\\ \text{Specificity}=\frac{\text{TN}}{\text{TN}+\text{FP}}\times \text{100}\\ \text{Accuracy}=\frac{\text{TP}+\text{TN}}{\text{TP}+\text{TN}+\text{FP}+\text{FN}}\times \text{100}\\ \text{MCC}=\frac{\text{(TP}\times \text{TN)}-\text{(FP}\times \text{FN)}}{\sqrt{\text{(TP}+\text{FP)(TP}+\text{FN)(TN}+\text{FP)(TN}+\text{FN)}}} \end{array}$$

Where TP and TN are correctly predicted positive and negative examples; FP and FN are falsely predicted positive and negative examples. MCC is a Matthew's correlation coefficient, which consider over and under prediction; MCC *1 *is regarded as a perfect prediction, whereas *0 *is regarded as random prediction.

#### One-versus-the-rest (*1-v-r*)

The prediction of substrate specificity of isoforms is a multi-class classification problem, where as SVM is a binary classifier. In order to handle this problem, we developed five models corresponding to five isoforms used in this study, one SVM model for each isoform. For example for developing a SVM model for CYP3A4, we consider substrates of CYP3A4 as positive examples and substrates of the rest of the isoforms as negative examples. Similarly for developing model for CYP2D6, substrates of CYP2D6 used as positive examples and substrates of the rest of the isoforms as negative examples.

#### Single Label Prediction

In case of single label prediction, we predict single best isoform responsible for metabolizing a drug/substrate. In other words for a substrate, we predict best isoform for this substrate. Following steps are performed in order to implement this single label prediction; i) one model for each isoform is developed on the training dataset (using *1-v-r*), ii) specificity of a substrate for each isoform is calculated using above models and iii) a isoform having highest specificity (SVM Score) for substrate is labeled as predicted isoform. In this type of prediction, only one class/label is assigned for a substrate. In order to assess the performance of these models we compute two parameters called average accuracy and overall accuracy. In case of average accuracy, we compute average of accuracy for five isoforms (mean of five accuracies). In case of overall accuracy, we compute overall percentage of correctly predicted substrates.

#### Multi-Label Prediction

It has been shown in previous studies that a drug can be metabolized by more than one CYP isoform. In our independent dataset we have substrates, which can be metabolized by more than one isoforms. Thus method has been developed to predict multiple labels/isoforms for a substrate. As described in one-versus-the-rest (*1-v-r*) section, five models have been developed (one for each isoform) and a default threshold was calculated for each model. In this study, a default threshold is a SVM score where performance of model is best in terms of MCC and difference between sensitivity and specificity is minimum. In order to predict isoform specificity of a substrate, we have computed SVM score of the substrate using each SVM model developed for five isoforms. If SVM score is more than the default threshold for an isoform then that isoform is assigned as metabolizing the substrate. It is possible that more than one isoforms are predicted as metabolizing isoform for a substrate. This type of prediction is called multi label prediction.

### SVM Algorithm

In the present study a highly successful machine learning technique, support vector machine (SVM) has been used for the prediction of isoform specificity. SVM is based on the structural risk minimization principle from statistical learning theory [20]. The whole theory of SVM can be simply described as follows: searching an optimal hyper-plane satisfies the request of classification, and then use a certain algorithm to make the margin of the separation beside the optimal hyper-plane maximum while ensuring the accuracy of correct classification [20,21]. SVM_light software package has been used to develop SVM based QSAR models. This software is freely downloaded from http://www.cs.cornell.edu/People/tj/svm_light/. In this study we tried various kernels like Radial Basis Function (RBF), polynomial and linear kernel in order to achieve best performance. The performance of models was optimized using systematic variation of different SVM parameters and kernels.

### Models using Weka

WEKA is a very popular and reliable package which is frequently used in Bioinformatics and QSAR studies. We used Weka version 3.6.0 [22] for developing different models, which is a collection of machine-learning algorithms. It supports several standard data mining tasks, data pre-processing, clustering, classification, regression, visualization, and feature selection. Here we used statistical and machine learning techniques implemented in Weka to predict the isoform specificity of CYP such as: (1) *Random Forest algorithm*: This is a meta-learner comprised of many individual trees, was designed to operate quickly over large datasets and more importantly to be diverse by using random samples to build each tree in the forest [23]; *SMOReg algorithm: *Sequential Minimization Optimization (SMO) [24,25] is a new algorithm for training SVM. This implementation globally replaced all missing values and transformed nominal attributes into binary ones. It also normalized all attributes by default; (3) *Rotation Forest algorithm: *This is a new classifiers for constructing an ensemble of trees using random subspaces and principal components transformation applied to the input data [26]; (4) *Simple logistic Algorithm*: This method build a logistic regression model using LogitBoost fitting (includes simple linear regression per attribute), incorporates attribute selection by fitting simple regression function in LogitBoost [27]; (5) *BayesNet algorithm*: A BayesNet is a probabilistic graphical model which represents a set of random variables and their conditional independencies via a directed acyclic graph (DAG). In this study BayesNet represents the probabilistic relationships between different CYP isoforms and their molecular descriptors [28,29]; (6) *REPTree*: This algorithm build model on the basis of decision/regression tree using information gain/feature reduction and prunes it using reduced -error pruning (with backfitting). This method considers all the attributes and missing value are dealt with by splitting the corresponding instances into pieces; (7) *RBF Network *: This method apply k-means clustering to find the basis functions within each class. The logistics regression's optimization algorithm automatically determines the coefficients for each variable. The magnitude of the coefficients are an indication to their relative importance for predicting the class [30]; (8) *Multilayer perceptron*: This is a feed forward artificial neural network model that maps sets of input data onto a set of appropriate output. It is a modification of the standard linear perceptron in that it uses three or more layers of neurons (nodes) with nonlinear activation functions, and is more powerful than the perceptron in that it can distinguish data that is not linearly separable, or separable by a hyperplane [31]; (9) *J48-IB1*: This is a based on the decision tree learning algorithm J48. A leaf may contain a simple nearest neighbor classifier [32] using one neighbor (i.e., IB1, in the terminology of Aha *et al*. [33]); (10) *NaiveBayes*: This is a simple probabilistic classifier. It assumes that every feature related to a class is independent of each other [34]. This algorithm implements Bayesian classification based on Bayes theorem of conditional probability. The theorem is used to estimate the probability of an example belonging to each of the possible classes of a classification problem [35]; (11) *KStar*: This is an instance-based classifier. In this algorithm a classification is based upon the class of those training instances similar to it, as determined by some similarity function [36] and (12) *Logistic Regression*: This algorithm use multinomial logistic regression model with a ridge estimator for classification [37].

## Results

### Performance on Main Dataset

The main dataset consists of total 216 substrates which can be metabolized by one of the five CYP isoforms used in this study. This dataset have 111, 47, 29, 20 and 19 molecules metabolized through isoform CYP 3A4, 2D6, 1A2, 2C9 and 2C19 respectively. As described in materials and method that prediction of isoform is a multi class classification as there are total five isoforms. SVM is a binary classifier which predict a instance positive or negative (yes or no). Thus we used one-versus-the-rest (*1-v-r*) SVM approach where one model per isoform has been developed. All models were developed using 41 selected attributes/descriptors of substrate, as described in materials and method section.

#### SVM based Model for each Isoform

First we have developed SVM model for isoform CYP3A4 using SVM_light package. The dataset for this isoform consists of 111 positive (substrates of CYP3A4) and 105 negative (rest of the substrates) examples. We have developed various models using different SVM parameters and evaluated each model using five-fold cross-validation technique. The performance of best model CYP3A4 is shown in Table 1. A high performance (MCC 0.63 with accuracy 81.42%) was achieved for this CYP3A4 isoform. Similarly model was developed for isoform CYP2D6 where dataset consists of 47 positive (substrates of CYP2D6) and 169 negative (rest of the substrates) examples. As shown in Table 1, we achieved best MCC 0.54 and accuracy 81.24% for isoform CYP2D6. Similarly, models were developed for remaining isoform used in this study, positive and negative examples used for each isoform is shown by POE and NEE columns of Table 1. In summary, we have achieved MCC 0.63, 0.54, 0.49, 0.40 and 0.15 for isoform CYP 3A4, 2D6, 1A2, 2C9 and 2C19 respectively. As shown in Table 1, the performance of a model also depends on number of positive examples. The model developed for CYP3A4 is best as it have nearly equal number of positive and negative examples.

**Table 1:** Performance of SVM models developed for different CYP isoforms, all models evaluated using fivefold cross-validation technique

Isoforms	POE*	NEE**	Sensitivity	Specificity	Accuracy (%)	MCC
CYP3A4	111	105	81.08	81.74	81.42	0.63
CYP2D6	47	169	74.47	83.24	81.24	0.54
CYP1A2	29	187	79.31	83.76	83.19	0.49
CYP2C9	20	196	70.00	85.92	84.51	0.40
CYP2C19	19	197	52.63	72.46	70.80	0.15

POE*: Positive Examples

NEE**: Negative Examples

Thres*: Threshold (Cutoff Value)

#### Single Label Prediction

In above section, we have demonstrated the performance of individual models developed for each isoform. In reality, a user normally would like to know the best metabolizing isoform for their substrate or drug molecule. Thus there is a need to predict single label/isoform for a substrate. In order to achieve single label prediction, first individual SVM models were trained on training dataset as described above. This way we got five models, one model for each isoform. Secondly SVM score was calculated for each substrate in test dataset using five models. In the third step, five SVM score of a substrate were compared to detect isoform having highest SVM score. Finally a metabolizing isoform was assigned for a substrate based on the highest SVM score. It is important to note that we are predicting best isoform for a compound. This does not mean that the compound is non-substrate for other isoforms. It is possible that this compound may be metabolized by other isoform. In order to access the performance, percent of correctly identified substrate of each isoform is computed, which is shown as accuracy (Table 2). We have achieved an accuracy of 78.76%, 83.19%, 87.61%, 91.15% and 89.38% for isoform CYP 3A4, 2D6, 1A2, 2C9 and 2C19 respectively, when evaluated using fivefold cross-validation technique. Ideally one need to evaluate the performance of models using LOOCV technique, in this study fivefold cross-validation technique has been used to save the computational time. In order to see the effect on performance, we have also evaluated the model using LOOCV. As shown in Table 2, the overall accuracy of models improved marginally from 82.81% to 83.58% in case of LOOCV. The average accuracy has also increased slightly from 86.02% to 86.20%.

**Table 2:** Percent of correctly predicted substrates (accuracy) belongs to different CYP isoforms where only single isoform was predicted for each substrate/molecule

CYP Isoform	Accuracy (percent)	Accuracy (percent)
	(5 fold CV)	(LOOCV)
CYP 1A2	78.76	80.53
CYP2C9	83.19	82.74
CYP2C19	87.61	84.96
CYP2D6	91.15	91.15
CYP3A4	89.38	91.59
**Average Accuracy**	**86.02**	**86.20**
**Overall Accuracy**	**82.81**	**83.58**

### WEKA based Model

WEKA is powerful software that allows users to develop models using various techniques [22]. We developed single label prediction models, as described above using various machine learning techniques. The performance in term of overall accuracy of the best model for each algorithm is shown in Table 3. It was observed that RandomForest performed best among SMOreg, Random Forest, Simple logostic, BayesNet, REPTree, RBF network, Multilayer perceptron, NaiveBayes, Logistic equation and tree based IB1 & Kstar. Though RandomForest of WEKA performed better than any other algorithm of WEKA but its overall accuracy 69.47% was lower than overall accuracy 82.81% of SVM_light. All models were evaluated using fivefold cross-validation technique.

**Table 3:** Overall accuracy achieved on main dataset, developed using different WEKA methods. Single label was predicted for each substrate and performance evaluated using five-fold cross-validation techniques

Methods	Overall accuracy (percent)
Random Forest	69.47
SMOreg	69.03
Rotation Forest	68.58
Simple logistic	66.37
BayesNet	65.04
REPTree	64.60
RBF network	64.16
Multilayer perceptron	62.39
IB1 (tree)	58.41
NaïveBayes	57.96
KStar (tree)	56.20
Logistic equation	51.77

### Performance on Independent dataset

Over optimization is one of the major drawbacks in cross-validation technique. Thus it is important to test the performance of a newly developed model on an independent dataset. As described in materials and method section, independent dataset consists of total 146 molecules. The number of molecules metabolized by isoform CYP 3A4, 2D6, 3A2, 2C9 and 2C19 are 92, 74, 41, 47 and 49 respectively. The molecules in this dataset can be metabolized by one or more than one CYP isoforms. Simply, this dataset also consists of multi label/isoform substrates; where as the main dataset exclusively consists of single label substrates. First SVM score was calculated for all molecules in independent dataset using SVM model of CYP 3A4 and all molecules having SVM score more than default threshold were predicted as molecule metabolized by CYP 3A4. Similarly, SVM score was calculated for remaining isoforms and predicted metabolizing isoform for each molecule. This is a multi label prediction where more than one isoform may be predicted for a molecule. Finally, we have computed percentage of correctly predicted substrate (accuracy) for each isoform. Out of 146 molecules, our models predicted 103 molecules correctly with an overall accuracy of 70.55%. As shown in Table 4, we have achieved average accuracy of 63.70% and the accuracy varies from 51.02% to 77.17% for different isoforms.

**Table 4:** Performance of SVM based isoform models on an Independent dataset. Multiple labels were assigned for substrates having SVM score more than default threshold for multiple isoform models

CYP Isoform	Accuracy (percent)
CYP3A4	77.17
CYP2D6	66.22
CYP1A2	68.30
CYP2C9	55.32
CYP2C19	51.02
**Average**	**63.70**

## Web server for Predicting Metabolizing

One of the major challenges for researchers working in the field of drug discovery is to predict the metabolizing isoform of a drug molecule. To the best of the author's knowledge there is no free software or web server for predicting metabolizing isoforms of a substrate, though number of methods have been developed in the past to predict substrate specificity [4-10]. Most of the powerful software packages commonly used for computing molecular descriptors are commercial and licensed for limited use. Thus it is not possible to use them for developing web server. One of the major aims of our group is to promote open source software [38].

In this study, we have also developed model using molecular descriptors calculated using following software packages; i) Chemistry Development Kit (CDK) a open source java library [39,40] and ii) a descriptors calculation software from Vlife [41]. Though Vlife is a commercial package but we bought the right to use its descriptors in our web server. Firstly we have computed 178 descriptors using CDK on our main dataset. Secondly, 26 best molecular descriptors were selected using WEKA based GreedyStepWise and genetic search approaches. These 26 descriptors were used to develop SVM models based on *1-v-r *approach. We have achieved overall accuracy of 81.42% on main dataset; single label was predicted for each substrate as described in above (single label prediction) section. The performance was evaluated using fivefold cross-validation technique. Similar approach was adopted for models based of Vlife descriptors and achieved maximum overall accuracy of 80.58% (Additional file 1: Table S9). As our models based on CDK descriptors perform better than models based on Vlife, we have developed and evaluated the performance of the rest of the models on CDK descriptors (Additional file 1: Table S10 - S11). The performance achieved using CDK descriptors is nearly the same as that was achieved using descriptors calculates using commercial software.

We have developed a server for predicting metabolizing CYP isoform of a drug molecule/substrate, based on SVM models developed using CDK descriptors. This server is installed on Linux (Red Hat) operating system. This is a user friendly web server, allows user to submit their molecule in mol2/sdf/smile format or by online drawing of molecule in JME editor. It also allows user to predict single or multiple label/isoform for a molecule. This server MetaPred is available free for public use from http://crdd.osdd.net/raghava/metapred/.

## Discussion

One of the most critical steps involved in discovering a new drug molecule is to understand its ADMET (absorption, distribution, metabolism, excretion and toxicity) properties. *In-silico *ADMET prediction may help to detect and eliminate compounds with poor pharmacokinetic properties at early stage of the drug development process. It is important to understand drug metabolism, as it is important component of ADMET. The different isoform of CYP involved in the phase I metabolism of drug molecules. It is important to know which isoform is responsible for the metabolism of a new drug molecule. The ability to predict sites and rates of metabolism of new substrates is useful in drug development, as well as in consideration of potential carcinogens and toxicants [42]. In this study, we have developed model for predicting substrate specificity five major CYP 3A4, 2D6, 1A2, 2C9 and 2C19 isoform. One of the major features of this study is clean and largest dataset. In a previous study, 146 substrates have been used to develop model for three isoforms; as per latest version of DrugBank a large number of molecules have specificity for more than one isoform. In our study we have used 216 drug molecules for five isoform, each molecule metabolized only by one isoform. It is important to have clean and large dataset for developing highly accurate isoform model. We have also evaluated the performance of our models on independent dataset extracted from DrugBank. Independent dataset consists of 146 molecules which interact with at least one of the five isoform; this dataset also includes molecules which can be metabolizing by more than one isoform. In summary, we have used largest possible data for our study from latest version of DrugBank.

One of the important factors which play a critical role in developing QSAR model is the number of molecular descriptors. As described in materials and method section, we have computed more than 2000 different types of descriptors encompassing almost all the properties of molecules. Another important step in development of models is the selection of best descriptors. Thus, all highly correlated, irrelevant molecular descriptor were removed; finally only the best 41 descriptors were used to develop QSAR models. Most of the powerful techniques commonly used for developing QSAR model (like SVM) are binary classifier whereas classification of isoform substrates is a multi class classification problem. In order to solve this problem, *1-v-r *approach has been used where five QSAR models have been developed for five isoform, one model for each isoform. One can develop QSAR model using number of techniques/algorithms. In this study first we have developed SVM based models, implemented using SVM_light package. As shown in Table 1, a reasonably high performance (MCC ≥ 0.4) was achieved for most of the isoform except for CYP 2C19. The performance for a model depends on number of substrates; unfortunately number of substrates is very small for most of the isoform. Though we have taken largest possible dataset but this dataset is not sufficient enough for developing very reliable models. These types of model can be improved significantly by training them on large experimental data.

It is important for a user to predict best CYP isoform responsible for metabolizing their drug molecule. Thus we have developed single-label prediction, where our method predicts single best isoform responsible for metabolizing a substrate/molecule. As shown in Table 2, reasonable high overall and average accuracy was achieved. It is also observed that the performance of fivefold cross-validation is as good as LOOCV. Thus in most of the study we have used fivefold cross-validation in order to save computational time. WEKA is one the powerful package which allows implementing a large number of algorithms, thus we have developed various QSAR model using these algorithms. As shown in Table 3, a reasonable overall accuracy was achieved for number of models. It was observed that SVM based QSAR models developed using SVM_light performed better than other models. Thus in rest of our study we have developed QSAR models for isoforms using SVM. It has been shown in the past that the cross-validation techniques suffer from over optimization, thus methods were also validated on an independent dataset. In case of evaluation of independent dataset, we have used multi label/isoform prediction approach where more than one isoform were also predicted for a substrate. It is because the independent dataset set also have substrates which are metabolized by more than one isoform. As shown in Table 4, we have obtained reasonably high accuracy on this dataset.

As shown in results section, SVM based models developed using 41 selected descriptors achieved very high accuracy. In order to compute these descriptors we have used commercial software. We have limited permissions to use these commercial due to license conditions. Thus we cannot provide service to community using these packages. In the past, number of methods has been developed for predicting substrate specificity of CYP isoforms but there is no web server for predicting isoform specificity. These studies have limited use for public. A large number of web servers have been developed in the field of bioinformatics, most of bioinformatics journal encourage authors to develop web server for public. In contrast there are very limited web servers. One of the objectives of our group is to encourage open source software. Recently we developed few servers in the field of chemoinformatics [43,44]. In order to develop web server for predicting metabolizing isoform of a drug molecule, we explore possibility. We found open source software CDK that allow calculating 178 molecular descriptors (one tenth of commercial descriptors). We have developed QSAR models based on SVM using 26 best descriptors of CDK. It has been observed that performance of models based on CDK descriptors is as good as we have achieved using descriptors obtained from commercial package. We developed a web server MetaPred for predicting isoform responsible for metabolizing a drug molecule, in order to promote open source software in chemoinformatics and to help the researcher working in the field of drug discovery.

## Conclusions

In this study, attempt has been made to predict substrate specificity of CYP 3A4, 2D6, 1A2, 2C9 and 2C19 isoforms using different approaches. We achieved an average accuracy more than 85% and overall accuracy more than 82% using single prediction approach. The dataset used in this study consists of latest and approved drug molecules. Thus the models developed in this study are more accurate and reliable, as they have been trained on large and clean dataset. First time models have been developed for five CYP isoforms. It has been demonstrated that models developed using CDK molecular descriptors are as good as models developed using descriptors calculated by commercial software. Based on this study we developed a web server MetaPred for predicting metabolizing isoform of a molecule. In long term this server will be useful for researchers working in the field of drug discovery. This study demonstrates that it is possible to develop free web servers in the field of chemoinformatics. This will encourage other researchers to develop web server for public use, which may lead to decrease the cost of discovering new drug molecules.

## Competing interests

The authors declare that they have no competing interests.

## Authors' contributions

NKM created dataset and developed the SVM models and revalidate these models. SA developed QSAR models using CDK and Vlife. SA also developed web server and revise the manuscript. GPSR conceived the project, coordinated it and refined the manuscript drafted by NKM and SA. All authors have read and approved this manuscript.

## Supplementary Material

Additional file 1 **Table S1: Main dataset of CYP2C9 substrate**. **Table-S2: **Main dataset of CYP2C19 substrate. **Table-S3: **Main dataset of CYP2D6 substrate. **Table-S4: **Main dataset of CYP3A4 substrate. **Table-S5: **Training dataset of CYP1A2 substrate. **Table-S6: **Independent Dataset (149 Molecule). **Table-S7: **List of 41Descriptors used to build SVM model.. **Table-S8: **Show 146 molecules, which was used by Terfloth et al, and found CYP biotransformation of only 126 molecules was reported in DrugBank. Out of these, 63 molecules metabolized with more than one CYP isoform.. **Table S9: **Performance of SVM models developed on CDK and Vlife descriptors, models evaluate using fivefold cross-validated technique. Prediction is based on single label prediction.. **Table S10: **Percent of correctly predicted substrates (Accuracy) belongs to different CYP isoforms where only single isoform was predicted for each substrate/molecule. **Table S11**: Performance of SVM models developed for different CYP isoforms, all models evaluated using fivefold cross-validation technique. Click here for file
