# Supplementary material for: Prediction of cytochrome P450 isoform responsible for metabolizing a drug molecule
Source: BMC Pharmacol. 2010 Jul 16;10:8. doi: 10.1186/1471-2210-10-8 (PMC2912882; doi:10.1186/1471-2210-10-8)
Supplement: Additional file 1 — Table S1: Main dataset of CYP2C9 substrate. Table-S2: Main dataset of CYP2C19 substrate. Table-S3: Main dataset of CYP2D6 substrate. Table-S4: Main dataset of CYP3A4 substrate. Table-S5: Training dataset of CYP1A2 substrate. Table-S6: Independent Dataset (149 Molecule). Table-S7: List of 41Descriptors used to build SVM model.. Table-S8: Show 146 molecules, which was used by Terfloth et al, and found CYP biotransformation of only 126 molecules was reported in DrugBank. Out of these, 63 molecules metabolized with more than one CYP isoform.. Table S9: Performance of SVM models developed on CDK and Vlife descriptors, models evaluate using fivefold cross-validated technique. Prediction is based on single label prediction.. Table S10: Percent of correctly predicted substrates (Accuracy) belongs to different CYP isoforms where only single isoform was predicted for each substrate/molecule. Table S11: Performance of SVM models developed for different CYP isoforms, all models evaluated using fivefold cross-validation technique. [file 1471-2210-10-8-S1.DOC]

# Additional File 1

Prediction of cytochrome P450 isoform responsible

for metabolizing a drug molecule

### Nitish K Mishra, Sandhya Agarwal and Gajendra PS Raghava*

Institute of Microbial Technology

Chandigarh, India

**Table S1:** Main dataset of CYP2C9 substrate

| **CYP2C9 Isofrom Substrate (20 Molecule)** | | |
| --- | --- | --- |
| **DB00175** | CYP2C9 | Pravastatin |
| **DB00177** | CYP2C9 | Valsartan |
| **DB00203** | CYP2C9 | Sildenafil |
| **DB00222** | CYP2C9 | Glimepiride |
| **DB00469** | CYP2C9 | Tenoxicam |
| **DB00472** | CYP2C9 | Fluoxetine |
| **DB00617** | CYP2C9 | Paramethadione |
| **DB00712** | CYP2C9 | Flurbiprofen |
| **DB00749** | CYP2C9 | Etodolac |
| **DB00784** | CYP2C9 | Mefenamic acid |
| **DB00814** | CYP2C9 | Meloxicam |
| **DB00821** | CYP2C9 | Carprofen |
| **DB00870** | CYP2C9 | Suprofen |
| **DB00916** | CYP2C9 | Metronidazole |
| **DB00946** | CYP2C9 | Phenprocoumon |
| **DB01015** | CYP2C9 | Sulfamethoxazole |
| **DB01029** | CYP2C9 | Irbesartan |
| **DB01050** | CYP2C9 | Ibuprofen |
| **DB01067** | CYP2C9 | Glipizide |
| **DB01283** | CYP2C9 | Lumiracoxib |

| **CYP2C19 Isofrom Substrate (19 Molecule)** | | |
| --- | --- | --- |
| **DB00231** | CYP2C19 | Temazepam |
| **DB00273** | CYP2C19 | Topiramate |
| **DB00328** | CYP2C19 | Indomethacin |
| **DB00338** | CYP2C19 | Omeprazole |
| **DB00349** | CYP2C19 | Clobazam |
| **DB00395** | CYP2C19 | Carisoprodol |
| **DB00420** | CYP2C19 | Promazine |
| **DB00444** | CYP2C19 | Teniposide |
| **DB00546** | CYP2C19 | Adinazolam |
| **DB00665** | CYP2C19 | Nilutamide |
| **DB00738** | CYP2C19 | Pentamidine |
| **DB00776** | CYP2C19 | Oxcarbazepine |
| **DB00794** | CYP2C19 | Primidone |
| **DB00927** | CYP2C19 | Famotidine |
| **DB00949** | CYP2C19 | Felbamate |
| **DB01041** | CYP2C19 | Thalidomide |
| **DB01060** | CYP2C19 | Amoxicillin |
| **DB01174** | CYP2C19 | Phenobarbital |
| **DB01544** | CYP2C19 | Flunitrazepam |
|  |  |  |

**Table-S2:** Main dataset of CYP2C19 substrate

| **CYP2D6 Isofrom Substrate (47 Molecule)**  **Table-S3:** Main dataset of CYP2D6 substrate | | | |
| --- | --- | --- | --- |
| **DB00182** | CYP2D6 | Amphetamine | |
| **DB00193** | CYP2D6 | Tramadol | |
| **DB00264** | CYP2D6 | Metoprolol | |
| **DB00285** | CYP2D6 | Venlafaxine | |
| **DB00289** | CYP2D6 | Atomoxetine | |
| **DB00295** | CYP2D6 | Morphine | |
| **DB00318** | CYP2D6 | Codeine | |
| **DB00335** | CYP2D6 | Atenolol | |
| **DB00373** | CYP2D6 | Timolol | |
| **DB00497** | CYP2D6 | Oxycodone | |
| **DB00514** | CYP2D6 | Dextromethorphan | |
| **DB00521** | CYP2D6 | Carteolol | |
| **DB00557** | CYP2D6 | Hydroxyzine | |
| **DB00575** | CYP2D6 | Clonidine | |
| **DB00612** | CYP2D6 | Bisoprolol | |
| **DB00623** | CYP2D6 | Fluphenazine | |
| **DB00647** | CYP2D6 | Propoxyphene | |
| **DB00656** | CYP2D6 | Trazodone | |
| **DB00672** | CYP2D6 | Chlorpropamide | |
| **DB00699** | CYP2D6 | Nicergoline | |
| **DB00715** | CYP2D6 | Paroxetine | |
| **DB00726** | CYP2D6 | Trimipramine | |
| **DB00734** | CYP2D6 | Risperidone | |
| **DB00805** | CYP2D6 | Minaprine | |
| **DB00850** | CYP2D6 | Perphenazine | |
| **DB00857** | CYP2D6 | Terbinafine | |
| **DB00866** | CYP2D6 | Alprenolol | |
| **DB00914** | CYP2D6 | Phenformin | |
| **DB00933** | CYP2D6 | Mesoridazine | |
| **DB00934** | CYP2D6 | Maprotiline | |
| **DB00960** | CYP2D6 | Pindolol |  |
| **DB01035** | CYP2D6 | Procainamide | |
| **DB01036** | CYP2D6 | Tolterodine | |
| **DB01069** | CYP2D6 | Promethazine | |
| **DB01071** | CYP2D6 | Mequitazine | |
| **DB01074** | CYP2D6 | Perhexiline | |
| **DB01142** | CYP2D6 | Doxepin |  |
| **DB01191** | CYP2D6 | Dexfenfluramine | |
| **DB01193** | CYP2D6 | Acebutolol | |
| **DB01195** | CYP2D6 | Flecainide | |
| **DB01197** | CYP2D6 | Captopril |  |
| **DB01210** | CYP2D6 | Levobunolol | |
| **DB01228** | CYP2D6 | Encainide | |
| **DB01233** | CYP2D6 | Metoclopramide | |
| **DB01403** | CYP2D6 | Methotrimeprazine | |
| **DB04840** | CYP2D6 | Debrisoquin | |
| **DB08148** | CYP2D6 | Mianserin |  |

**Table-S4:** Main dataset of CYP3A4 substrate

| **CYP3A4 Isofrom Substrate (111 Molecule)** | | |
| --- | --- | --- |
| **DB00180** | CYP3A4 | Flunisolide |
| **DB00196** | CYP3A4 | Fluconazole |
| **DB00204** | CYP3A4 | Dofetilide |
| **DB00216** | CYP3A4 | Eletriptan |
| **DB00240** | CYP3A4 | Alclometasone |
| **DB00246** | CYP3A4 | Ziprasidone |
| **DB00251** | CYP3A4 | Terconazole |
| **DB00270** | CYP3A4 | Isradipine |
| **DB00280** | CYP3A4 | Dispyramide |
| **DB00294** | CYP3A4 | Etonogestrel |
| **DB00297** | CYP3A4 | Bupivacalne |
| **DB00307** | CYP3A4 | Bexarotene |
| **DB00309** | CYP3A4 | Vindesine |
| **DB00320** | CYP3A4 | Dihydroergotamine |
| **DB00324** | CYP3A4 | Fluorometholone |
| **DB00337** | CYP3A4 | Pimecrolimus |
| **DB00346** | CYP3A4 | Alfuzosin |
| **DB00361** | CYP3A4 | Vinorelbine |
| **DB00381** | CYP3A4 | Amlodipine |
| **DB00393** | CYP3A4 | Nimodiplne |
| **DB00398** | CYP3A4 | Sorafenlb |
| **DB00401** | CYP3A4 | Nisoldipine |
| **DB00404** | CYP3A4 | Alprazolam |
| **DB00425** | CYP3A4 | Zolpidem |
| **DB00443** | CYP3A4 | Betamethasone |
| **DB00445** | CYP3A4 | Epirubicin |
| **DB00451** | CYP3A4 | Levothyroxlne |
| **DB00490** | CYP3A4 | Buspirone |
| **DB00518** | CYP3A4 | Albendazole |
| **DB00528** | CYP3A4 | Lercanidipine |
| **DB00530** | CYP3A4 | Erlotinib |
| **DB00539** | CYP3A4 | Toremifene |
| **DB00542** | CYP3A4 | Benazepril |
| **DB00570** | CYP3A4 | Vinblastine |
| **DB00584** | CYP3A4 | Enalapril |
| **DB00588** | CYP3A4 | Fluticasone Propionate |
| **DB00591** | CYP3A4 | Fluocinolone Acetonide |
| **DB00604** | CYP3A4 | Cisapride |
| **DB00615** | CYP3A4 | Rifabutin |
| **DB00619** | CYP3A4 | Imatinib |
| **DB00635** | CYP3A4 | Prednisone |
| **DB00637** | CYP3A4 | Astemizole |
| **DB00643** | CYP3A4 | Mebendazole |
| **DB00646** | CYP3A4 | Nystatin |
| **DB00663** | CYP3A4 | Flumethasone Pivalate |
| **DB00696** | CYP3A4 | Ergotamine |
| **DB00700** | CYP3A4 | Eplerenone |
| **DB00701** | CYP3A4 | Amprenavir |
| **DB00717** | CYP3A4 | Norethindrone |
| **DB00741** | CYP3A4 | Hydrocortisone |
| **DB00764** | CYP3A4 | Mometasone |
| **DB00769** | CYP3A4 | Hydrocortamate |
| **DB00773** | CYP3A4 | Etoposide |
| **DB00778** | CYP3A4 | Roxithromycin |
| **DB00813** | CYP3A4 | Fentanyl |
| **DB00820** | CYP3A4 | Tadalafil |
| **DB00836** | CYP3A4 | Loperamide |
| **DB00846** | CYP3A4 | Flurandrenolide |
| **DB00860** | CYP3A4 | Prednisolone |
| **DB00862** | CYP3A4 | Vardenafil |
| **DB00872** | CYP3A4 | Conivaptan |
| **DB00877** | CYP3A4 | Sirolimus |
| **DB00881** | CYP3A4 | Quinapril |
| **DB00883** | CYP3A4 | Isosorbide Dinitrate |
| **DB00897** | CYP3A4 | Triazolam |
| **DB00906** | CYP3A4 | Tiagabine |
| **DB00907** | CYP3A4 | Cocaine |
| **DB00909** | CYP3A4 | Zonisamide |
| **DB00911** | CYP3A4 | Tinidazole |
| **DB00932** | CYP3A4 | Tipranavir |
| **DB00938** | CYP3A4 | Salmeterol |
| **DB00947** | CYP3A4 | Fulvestrant |
| **DB00950** | CYP3A4 | Fexofenadine |
| **DB00959** | CYP3A4 | Methylprednisolone |
| **DB00962** | CYP3A4 | Zaleplon |
| **DB00990** | CYP3A4 | Exemestane |
| **DB00997** | CYP3A4 | Doxorubicin |
| **DB01008** | CYP3A4 | Busulfan |
| **DB01020** | CYP3A4 | Isosorbide Mononitrate |
| **DB01023** | CYP3A4 | Felodipine |
| **DB01047** | CYP3A4 | Fluocinonide |
| **DB01054** | CYP3A4 | Nitrendiplne |
| **DB01062** | CYP3A4 | Oxybutynin |
| **DB01068** | CYP3A4 | Clonazepam |
| **DB01072** | CYP3A4 | Atazanavir |
| **DB01076** | CYP3A4 | Atorvastatin |
| **DB01105** | CYP3A4 | Sibutramine |
| **DB01126** | CYP3A4 | Dutasteride |
| **DB01157** | CYP3A4 | Trimetrexate |
| **DB01184** | CYP3A4 | Domperidone |
| **DB01190** | CYP3A4 | Clindamycin |
| **DB01200** | CYP3A4 | Bromocriptine |
| **DB01206** | CYP3A4 | Lomustine |
| **DB01215** | CYP3A4 | Estazolam |
| **DB01222** | CYP3A4 | Budesonide |
| **DB01227** | CYP3A4 | Levomethadyl Acetate |
| **DB01234** | CYP3A4 | Dexamethasone |
| **DB01244** | CYP3A4 | Bepridil |
| **DB01254** | CYP3A4 | Dasatinib |
| **DB01256** | CYP3A4 | Retapamulin |
| **DB01258** | CYP3A4 | Aliskiren |
| **DB01263** | CYP3A4 | Posaconazole |
| **DB01264** | CYP3A4 | Darunavir |
| **DB01268** | CYP3A4 | Sunitinib |
| **DB01319** | CYP3A4 | Fosamprenavir |
| **DB01395** | CYP3A4 | Drospirenone |
| **DB01411** | CYP3A4 | Pranlukast |
| **DB01591** | CYP3A4 | Solifenacin |
| **DB01601** | CYP3A4 | Lopinavir |
| **DB01628** | CYP3A4 | Etoricoxib |
| **DB04835** | CYP3A4 | Maraviroc |
| **DB04839** | CYP3A4 | Cyproterone |

**Table-S5:** Training dataset of CYP1A2 substrate

| **CYP1A2 Isofrom Substrate (29 Molecule)** | | |
| --- | --- | --- |
| **DB00201** | CYP1A2 | Caffeine |
| **DB00261** | CYP1A2 | Anagrelide |
| **DB00262** | CYP1A2 | Carmustine |
| **DB00268** | CYP1A2 | Ropinirole |
| **DB00296** | CYP1A2 | Ropivacaine |
| **DB00315** | CYP1A2 | Zolmitriptan |
| **DB00316** | CYP1A2 | Acetaminophen |
| **DB00334** | CYP1A2 | Olanzapine |
| **DB00382** | CYP1A2 | Tacrine |
| **DB00467** | CYP1A2 | Enoxacin |
| **DB00475** | CYP1A2 | Chlordiazepoxide |
| **DB00487** | CYP1A2 | Pefloxacin |
| **DB00499** | CYP1A2 | Flutamide |
| **DB00537** | CYP1A2 | Ciprofloxacin |
| **DB00544** | CYP1A2 | Fluorouracil |
| **DB00571** | CYP1A2 | Propranolol |
| **DB00697** | CYP1A2 | Tizanidine |
| **DB00740** | CYP1A2 | Riluzole |
| **DB00787** | CYP1A2 | Aciclovir |
| **DB00924** | CYP1A2 | Cyclobenzaprine |
| **DB00978** | CYP1A2 | Lomefloxacin |
| **DB00980** | CYP1A2 | Ramefloxacin |
| **DB00998** | CYP1A2 | Frovatriptan |
| **DB01059** | CYP1A2 | Norfloxacin |
| **DB01094** | CYP1A2 | Hesperetin |
| **DB01097** | CYP1A2 | Leflunomide |
| **DB01137** | CYP1A2 | Levofloxacin |
| **DB01165** | CYP1A2 | Ofloxacin |
| **DB01367** | CYP1A2 | Rasagiline |

**Table-S6: Independent Dataset (149 Molecule)**

| **Drugbank ID** | **Generic Name** |
| --- | --- |
| **DB00176** | Fluvoxamine |
| **DB00185** | Cevimeline |
| **DB00208** | Ticlopidine |
| **DB00213** | Pantoprazole |
| **DB00214** | Torasemide |
| **DB00215** | Citalopram |
| **DB00220** | Nelfinavir |
| **DB00224** | Indinavir |
| **DB00227** | Lovastatin |
| **DB00234** | Reboxetine |
| **DB00238** | Nevirapine |
| **DB00243** | Ranolazine |
| **DB00250** | Dapsone |
| **DB00252** | Phenytoin |
| **DB00277** | Theophylline |
| **DB00281** | Lidocaine |
| **DB00304** | Desogestrel |
| **DB00317** | Gefitinib |
| **DB00321** | Amitriptyline |
| **DB00327** | Hydromorphone |
| **DB00332** | Ipratropium |
| **DB00333** | Methadone |
| **DB00342** | Terfenadine |
| **DB00343** | Diltiazem |
| **DB00356** | Chlorzoxazone |
| **DB00363** | Clozapine |
| **DB00365** | Grepafloxacin |
| **DB00379** | Mexiletine |
| **DB00396** | Progesterone |
| **DB00402** | Eszopiclone |
| **DB00412** | Rosiglitazone |
| **DB00433** | Prochlorperazine |
| **DB00448** | Lansoprazole |
| **DB00454** | Meperidine |
| **DB00455** | Loratadine |
| **DB00458** | Imipramine |
| **DB00468** | Quinine |
| **DB00470** | Marinol |
| **DB00471** | Montelukast |
| **DB00476** | Duloxetine |
| **DB00477** | Chlorpromazine |
| **DB00482** | Celecoxib |
| **DB00496** | Darifenacin |
| **DB00502** | Haloperidol |
| **DB00503** | Ritonavir |
| **DB00506** | Norgestrel |
| **DB00527** | Dibucaine |
| **DB00532** | Mephenytoin |
| **DB00540** | Nortriptyline |
| **DB00541** | Vincristine |
| **DB00549** | Zafirlukast |
| **DB00559** | Bosentan |
| **DB00564** | Carbamazepine |
| **DB00582** | Voriconazole |
| **DB00586** | Diclofenac |
| **DB00593** | Ethosuximide |
| **DB00603** | Medroxyprogesterone |
| **DB00613** | Amodiaquine |
| **DB00622** | Nicardipine |
| **DB00624** | Testosterone |
| **DB00625** | Efavirenz |
| **DB00636** | Clofibrate |
| **DB00641** | Simvastatin |
| **DB00655** | Estrone |
| **DB00661** | Verapamil |
| **DB00674** | Galantamine |
| **DB00675** | Tamoxifen |
| **DB00678** | Losartan |
| **DB00679** | Thioridazine |
| **DB00682** | Warfarin |
| **DB00683** | Midazolam |
| **DB00705** | Delavirdine |
| **DB00730** | Thiabendazole |
| **DB00731** | Nateglinide |
| **DB00736** | Esomeprazole |
| **DB00744** | Zileuton |
| **DB00745** | Modafinil |
| **DB00757** | Dolasetron |
| **DB00758** | Clopidogrel |
| **DB00762** | Irinotecan |
| **DB00783** | Estradiol |
| **DB00802** | Alfentanil |
| **DB00829** | Diazepam |
| **DB00830** | Phenmetrazine |
| **DB00834** | Mifepristone |
| **DB00843** | Donepezil |
| **DB00849** | Methylphenobarbital |
| **DB00863** | Ranitidine |
| **DB00864** | Tacrolimus |
| **DB00865** | Benzphetamine |
| **DB00889** | Granisetron |
| **DB00904** | Ondansetron |
| **DB00908** | Quinidine |
| **DB00918** | Almotriptan |
| **DB00921** | Buprenorphine |
| **DB00956** | Hydrocodone |
| **DB00967** | Desloratadine |
| **DB00969** | Alosetron |
| **DB00972** | Azelastine |
| **DB00976** | Telithromycin |
| **DB00977** | Ethinyl Estradiol |
| **DB00983** | Formoterol |
| **DB01002** | Levobupivacaine |
| **DB01006** | Letrozole |
| **DB01012** | Cinacalcet |
| **DB01018** | Guanfacine |
| **DB01026** | Ketoconazole |
| **DB01065** | Melatonin |
| **DB01075** | Diphenhydramine |
| **DB01095** | Fluvastatin |
| **DB01100** | Pimozide |
| **DB01104** | Sertraline |
| **DB01110** | Miconazole |
| **DB01114** | Chlorpheniramine |
| **DB01115** | Nifedipine |
| **DB01118** | Amiodarone |
| **DB01124** | Tolbutamide |
| **DB01129** | Rabeprazole |
| **DB01131** | Proguanil |
| **DB01132** | Pioglitazone |
| **DB01136** | Carvedilol |
| **DB01149** | Nefazodone |
| **DB01151** | Desipramine |
| **DB01156** | Bupropion |
| **DB01166** | Cilostazol |
| **DB01167** | Itraconazole |
| **DB01173** | Orphenadrine |
| **DB01175** | Escitalopram |
| **DB01182** | Propafenone |
| **DB01192** | Oxymorphone |
| **DB01211** | Clarithromycin |
| **DB01216** | Finasteride |
| **DB01218** | Halofantrine |
| **DB01224** | Quetiapine |
| **DB01229** | Paclitaxel |
| **DB01232** | Saquinavir |
| **DB01238** | Aripiprazole |
| **DB01241** | Gemfibrozil |
| **DB01242** | Clomipramine |
| **DB01259** | Lapatinib |
| **DB01261** | Sitagliptin |
| **DB01267** | Paliperidone |
| **DB01274** | Arformoterol |
| **DB01320** | Fosphenytoin |
| **DB01361** | Troleandomycin |
| **DB01409** | Tiotropium |
| **DB01410** | Ciclesonide |
| **DB01418** | Acenocoumarol |
| **DB06144** | Sertindole |

**Table-S7:** List of 41Descriptors used to build SVM model

| **Descriptor list** |
| --- |
| **Total Lipole** |
| **6-membered rings** |
| **VAMP Octupole ZZZ** |
| **VAMP Octupole ZZY** |
| **Group count for Amino** |
| **Group count for Ethyl** |
| **Lipole X Component** |
| **Molecular Surface Area** |
| **VAMP Polarization XZ** |
| **VAMP Quadpole XY** |
| **Dipole Moment Y Component** |
| **Lipole Z Component** |
| **Total Dipole Moment** |
| **VAMP Octupole XXZ** |
| **Molecular Refractivity** |
| **VAMP Ionizational Potential** |
| **NS (Number of sulfurs)** |
| **NDB (Number of Double bonds)** |
| **MOMH-4 (Ration of the first and second moments of inertia)** |
| **SHDW-1(Shadow area projected onto the XY plane)** |
| **SHDW-2(Shadow area projected onto the XZ plane)** |
| **SHDW-4 (Standardized SHDW-1-to factor out size contributions)** |
| **SRMN** |
| **STRA2** |
| **GEOM-3(Z-principal geometric moment)** |
| **GEOM-4(X/Y ratio)** |
| **MDEC-33(molecular distance edge between all tertiary carbons)** |
| **EMIN-1(minimum atomic e-state value)** |
| **EAVE-2 (average e-state value over all hydroatoms)** |
| **PND-3 (superpendentic index- only pendent nitrogens considered )** |
| **PND5 (superpendentic index- only pendent oxigens considered )** |
| **ENEG (Electronegativity 0.5*(HOMO+LUMO))** |
| **HARD (hardness 0.5*(HOMO~LUMO))** |
| **PCHGN** |
| **PCHGNH** |
| **PCHGPC** |
| **PCHGPN** |
| **PCHGPX** |
| **3SP2 (Double bound carbon bound to three other carbons)** |
| **4SP3(singly bound carbon bound to three other carbons)** |
| **CRB_LEADL** |

**Table-S8: Show 146 molecules, which was used by Terfloth et al, and found CYP biotransformation of only 126 molecules was reported in DrugBank. Out of these, 63 molecules metabolized with more than one CYP isoform.**

| **Generic Name** | **Terfloth et al Data** |  | **DrugBank Report** |  |  |  |  |  |  |  |
| --- | --- | --- | --- | --- | --- | --- | --- | --- | --- | --- |
| **Alfentanil** | CYP3A |  | CYP3A4 | CYP3A5 |  |  |  |  |  |  |
| **Alprazolam** | CYP3A |  | CYP3A4 |  |  |  |  |  |  |  |
| **Amiodarone** | CYP3A |  | CYP2C8 | CYP2C9 | CYP2D6 |  |  |  |  |  |
| **Amlodipine** | CYP3A |  | CYP3A4 |  |  |  |  |  |  |  |
| **Astemizole** | CYP3A |  | CYP3A4 |  |  |  |  |  |  |  |
| **Bepridil** | CYP3A |  | CYP3A4 |  |  |  |  |  |  |  |
| **Carbamazepine** | CYP3A |  | CYP3A4 | CYP2C8 | CYP2B6 |  |  |  |  |  |
| **Cisapride** | CYP3A |  | CYP3A4 |  |  |  |  |  |  |  |
| **Clarithromycin** | CYP3A |  | CYP3A4 | CYP2C19 |  |  |  |  |  |  |
| **Cocaine** | CYP3A |  | CYP3A4 |  |  |  |  |  |  |  |
| **Cyclosporin** | CYP3A |  | CYP3A5 |  |  |  |  |  |  |  |
| **Dexamethasone** | CYP3A |  | CYP3A4 |  |  |  |  |  |  |  |
| **Diltiazem** | CYP3A |  | CYP3A4 | CYP2C19 | CYP2D6 |  |  |  |  |  |
| **Disopyramide** | CYP3A |  | CYP3A4 |  |  |  |  |  |  |  |
| **Ergotamine** | CYP3A |  | CYP3A4 |  |  |  |  |  |  |  |
| **Erythromycin** | CYP3A |  | CYP3A4 |  |  |  |  |  |  |  |
| **Ethinylestradiol** | CYP3A |  | CYP3A4 | CYP2A6 |  |  |  |  |  |  |
| **Ethosuximide** | CYP3A |  | CYP3A4 | CYP2E1 |  |  |  |  |  |  |
| **Etoposide** | CYP3A |  | CYP3A4 |  |  |  |  |  |  |  |
| **Felodipine** | CYP3A |  | CYP3A4 |  |  |  |  |  |  |  |
| **Fentanyl** | CYP3A |  | CYP3A4 |  |  |  |  |  |  |  |
| **Finasteride** | CYP3A |  | CYP3A4 | CYP2C19 |  |  |  |  |  |  |
| **Fluconazole** | CYP3A |  | CYP3A4 | CYP2C9 |  |  |  |  |  |  |
| **Flutamide** | CYP3A |  | CYP1A2 |  |  |  |  |  |  |  |
| **Ifosfamide** | CYP3A |  | CYP2B6 |  |  |  |  |  |  |  |
| **Indinavir** | CYP3A |  | CYP3A4 | CYP3A5 |  |  |  |  |  |  |
| **Isradipine** | CYP3A |  | CYP3A4 |  |  |  |  |  |  |  |
| **Itraconazole** | CYP3A |  | CYP3A4 | CYP2D6 |  |  |  |  |  |  |
| **Ketoconazole** | CYP3A |  | CYP3A4 | CYP3A5 | CYP2C9 | CYP2C19 | CYP2C8 | CYP1A2 | CYP1A1 | CYP2D9 |
| **Lidocaine** | CYP3A |  | CYP1A2 | CYP2D6 |  |  |  |  |  |  |
| **Loratadine** | CYP3A |  | CYP3A4 | CYP2D6 | CYP2C19 |  |  |  |  |  |
| **Methadone** | CYP3A |  | CYP3A4 | CYP2B6 | CYP2C19 |  |  |  |  |  |
| **Methylprednisolone** | CYP3A |  | CYP3A4 |  |  |  |  |  |  |  |
| **Miconazole** | CYP3A |  | CYP2C9 | CYP2D6 |  |  |  |  |  |  |
| **Midazolam** | CYP3A |  | CYP3A5 | CYP2E1 | CYP3A4 | CYP2C9 | CYP2C8 | CYP2D6 | CYP2B6 |  |
| **Nefazodone** | CYP3A |  | CYP3A4 | CYP2D6 |  |  |  |  |  |  |
| **Nicardipine** | CYP3A |  | CYP2C19 | CYP3A4 | CYP2C9 | CYP2C8 | CYP2D6 |  |  |  |
| **Nifedipine** | CYP3A |  | CYP2E1 | CYP3A4 | CYP2D6 |  |  |  |  |  |
| **Nimodipine** | CYP3A |  | CYP3A4 |  |  |  |  |  |  |  |
| **Nisoldipine** | CYP3A |  | CYP3A4 |  |  |  |  |  |  |  |
| **Nitrendipine** | CYP3A |  | CYP3A4 |  |  |  |  |  |  |  |
| **Pimozide** | CYP3A |  | CYP3A4 | CYP1A2 |  |  |  |  |  |  |
| **Prednisolone** | CYP3A |  | CYP3A4 |  |  |  |  |  |  |  |
| **Quinine** | CYP3A |  | CYP3A4 | CYP3A5 | CYP2D6 | CYP1A1 |  |  |  |  |
| **Rapamycin** | CYP3A |  | NA |  |  |  |  |  |  |  |
| **Rifabutin** | CYP3A |  | CYP3A4 |  |  |  |  |  |  |  |
| **Ritonavir** | CYP3A |  | CYP3A4 | CYP2C9 | CYP2C19 | CYP2D6 | CYP2B6 |  |  |  |
| **Saquinavir** | CYP3A |  | CYP3A4 | CYP3A5 |  |  |  |  |  |  |
| **Sertraline** | CYP3A |  | CYP2C9 | CYP2C19 | CYP2B6 | CYP2D6 |  |  |  |  |
| **Simvastatin** | CYP3A |  | CYP3A4 | CYP2C8 |  |  |  |  |  |  |
| **Tacrolimus** | CYP3A |  | CYP3A4 | CYP3A5 |  |  |  |  |  |  |
| **Tamoxifen** | CYP3A |  | CYP3A4 | CYP2C9 | CYP2D6 |  |  |  |  |  |
| **Terfenadine** | CYP3A |  | CYP2C19 | CYP3A4 | CYP1A2 | CYP2C9 | CYP2D6 |  |  |  |
| **Testosterone** | CYP3A |  | CYP3A4 | CYP11A1 | CYP19 |  |  |  |  |  |
| **Triazolam** | CYP3A |  | CYP3A4 |  |  |  |  |  |  |  |
| **Verapamil** | CYP3A |  | CYP3A5 | CYP1A2 |  |  |  |  |  |  |
| **Vinblastine** | CYP3A |  | CYP3A4 |  |  |  |  |  |  |  |
| **Vincristine** | CYP3A |  | CYP314 | CYP3A5 |  |  |  |  |  |  |
| **Zidovudine** | CYP3A |  | NA |  |  |  |  |  |  |  |
| **Zolpidem** | CYP3A |  | CYP3A4 |  |  |  |  |  |  |  |
| **Codeine** | CYP2D6 |  | CYP2D6 |  |  |  |  |  |  |  |
| **Desipramine** | CYP2D6 |  | CYP2D6 | CYP2C19 |  |  |  |  |  |  |
| **Dexfenfluramine** | CYP2D6 |  | CYP2D6 |  |  |  |  |  |  |  |
| **Encainide** | CYP2D6 |  | CYP2D6 |  |  |  |  |  |  |  |
| **Flecainide** | CYP2D6 |  | CYP2D6 |  |  |  |  |  |  |  |
| **Fluoxetine** | CYP2D6 |  | CYP2C9 |  |  |  |  |  |  |  |
| **Fluvoxamine** | CYP2D6 |  | CYP2C19 | CYP1A2 | CYP2C9 | CYP2D6 | CYP1A1 |  |  |  |
| **Haloperidol** | CYP2D6 |  | CYP2D6 | CYP1A2 |  |  |  |  |  |  |
| **Hydrocodone** | CYP2D6 |  | CYP3A4 | CYP2D6 |  |  |  |  |  |  |
| **Maprotiline** | CYP2D6 |  | CYP2D6 |  |  |  |  |  |  |  |
| **Methamphetamine** | CYP2D6 |  | NA |  |  |  |  |  |  |  |
| **Metoprolol** | CYP2D6 |  | CYP2D6 |  |  |  |  |  |  |  |
| **Mexiletine** | CYP2D6 |  | CYP2D6 | CYP1A2 |  |  |  |  |  |  |
| **Nortriptyline** | CYP2D6 |  | CYP2D6 | CYP1A2 |  |  |  |  |  |  |
| **Oxycodone** | CYP2D6 |  | CYP2D6 |  |  |  |  |  |  |  |
| **Paroxetine** | CYP2D6 |  | CYP2D6 |  |  |  |  |  |  |  |
| **Perphenazine** | CYP2D6 |  | CYP2D6 |  |  |  |  |  |  |  |
| **Propafenone** | CYP2D6 |  | CYP1A2 | CYP2D6 |  |  |  |  |  |  |
| **Risperidone** | CYP2D6 |  | CYP2D6 |  |  |  |  |  |  |  |
| **Thioridazine** | CYP2D6 |  | CYP2D6 | CYP2C19 |  |  |  |  |  |  |
| **Timolol** | CYP2D6 |  | CYP2D6 |  |  |  |  |  |  |  |
| **Tramadol** | CYP2D6 |  | CYP2D6 |  |  |  |  |  |  |  |
| **Trazodone** | CYP2D6 |  | CYP2D6 |  |  |  |  |  |  |  |
| **Trimipramine** | CYP2D6 |  | CYP2D6 |  |  |  |  |  |  |  |
| **Venlafaxine** | CYP2D6 |  | CYP2D6 |  |  |  |  |  |  |  |
| **Diclofenac** | CYP2C9 |  | CYP2C19 | CYP1A2 | CYP2C8 | CYP2C9 | CYP2D6 |  |  |  |
| **Dronabinol** | CYP2C9 |  | CYP3A4 | CYP2C9 |  |  |  |  |  |  |
| **Flurbiprofen** | CYP2C9 |  | CYP2C9 |  |  |  |  |  |  |  |
| **Glimepiride** | CYP2C9 |  | CYP2C9 |  |  |  |  |  |  |  |
| **Ibuprofen** | CYP2C9 |  | CYP2C9 |  |  |  |  |  |  |  |
| **Indomethacin** | CYP2C9 |  | CYP2C19 |  |  |  |  |  |  |  |
| **Naproxen** | CYP2C9 |  | NA |  |  |  |  |  |  |  |
| **Phenytoin** | CYP2C9 |  | CYP3A5 | CYP2C19 | CYP2C8 | CYP2C9 | CYP2B6 |  |  |  |
| **Piroxicam** | CYP2C9 |  | NA |  |  |  |  |  |  |  |
| **Tolbutamide** | CYP2C9 |  | CYP2A6 | CYP2E1 | CYP2C19 | CYP1A2 | CYP2C8 | CYP2C9 | CYP2D6 |  |
| **Torsemide** | CYP2C9 |  | CYP2C8 | CYP2C9 |  |  |  |  |  |  |
| **Alpidem** | CYP3A |  | NA |  |  |  |  |  |  |  |
| **Azatadine** | CYP3A |  | NA |  |  |  |  |  |  |  |
| **Budesonide** | CYP3A |  | CYP3A4 |  |  |  |  |  |  |  |
| **Colchicine** | CYP3A |  | NA |  |  |  |  |  |  |  |
| **Cortisol** | CYP3A |  | CYP3A4 |  |  |  |  |  |  |  |
| **Cyclobenzaprine** | CYP3A |  | CYP1A2 |  |  |  |  |  |  |  |
| **Ebastine** | CYP3A |  | NA |  |  |  |  |  |  |  |
| **Enalapril** | CYP3A |  | CYP3A4 |  |  |  |  |  |  |  |
| **Gestodene** | CYP3A |  | NA |  |  |  |  |  |  |  |
| **Glibenclamide** | CYP3A |  | CYP2C9 |  |  |  |  |  |  |  |
| **Irinotecan** | CYP3A |  | CYP3A4 | CYP3A5 |  |  |  |  |  |  |
| **Levonorgestrel** | CYP3A |  | NA |  |  |  |  |  |  |  |
| **Lisuride** | CYP3A |  | NA |  |  |  |  |  |  |  |
| **Mifepristone** | CYP3A |  | CYP3A4 | CYP3A5 |  |  |  |  |  |  |
| **Primidone** | CYP3A |  | CYP2C19 |  |  |  |  |  |  |  |
| **Quercetin** | CYP3A |  | NA |  |  |  |  |  |  |  |
| **Quetiapine** | CYP3A |  | CYP3A4 | CYP2D6 |  |  |  |  |  |  |
| **Sertindole** | CYP3A |  | CYP3A4 | CYP2D6 |  |  |  |  |  |  |
| **Sulfidimidine** | CYP3A |  | NA |  |  |  |  |  |  |  |
| **Toremifene** | CYP3A |  | CYP3A4 |  |  |  |  |  |  |  |
| **Amiflamine** | CYP2D6 |  | NA |  |  |  |  |  |  |  |
| **Bufuralol** | CYP2D6 |  | NA |  |  |  |  |  |  |  |
| **Chlorpheniramine** | CYP2D6 |  | CYP3A4 | CYP2D6 |  |  |  |  |  |  |
| **Cinnarizine** | CYP2D6 |  | CYP2E1 |  |  |  |  |  |  |  |
| **Debrisoquine** | CYP2D6 |  | CYP2D6 |  |  |  |  |  |  |  |
| **Deprenyl** | CYP2D6 |  | CYP2C19 | CYP2B6 | CYP2D6 |  |  |  |  |  |
| **Flunarizine** | CYP2D6 |  | NA |  |  |  |  |  |  |  |
| **Fluphenazine** | CYP2D6 |  | CYP2D6 |  |  |  |  |  |  |  |
| **Indoramin** | CYP2D6 |  | NA |  |  |  |  |  |  |  |
| **Lobeline** | CYP2D6 |  | NA |  |  |  |  |  |  |  |
| **Methoxyphenamine** | CYP2D6 |  | NA |  |  |  |  |  |  |  |
| **Mianserin** | CYP2D6 |  | CYP2D6 |  |  |  |  |  |  |  |
| **Minaprine** | CYP2D6 |  | CYP2D6 |  |  |  |  |  |  |  |
| **Morphine** | CYP2D6 |  | CYP2D6 |  |  |  |  |  |  |  |
| **Perhexiline** | CYP2D6 |  | CYP2D6 |  |  |  |  |  |  |  |
| **Phenformin** | CYP2D6 |  | CYP2D6 |  |  |  |  |  |  |  |
| **Remoxipride** | CYP2D6 |  | AADC |  |  |  |  |  |  |  |
| **Sparteine** | CYP2D6 |  | NA |  |  |  |  |  |  |  |
| **Tropisetron** | CYP2D6 |  | NA |  |  |  |  |  |  |  |
| **Zuclopenthixol** | CYP2D6 |  | CYP2D6 |  |  |  |  |  |  |  |
| **Aceclofenac** | CYP2C9 |  | NA |  |  |  |  |  |  |  |
| **Fluvastatin** | CYP2C9 |  | CYP2C8 | CYP2C9 | CYP1A1 |  |  |  |  |  |
| **Lornoxicam** | CYP2C9 |  | NA |  |  |  |  |  |  |  |
| **Mefenamic acid** | CYP2C9 |  | CYP2C9 |  |  |  |  |  |  |  |
| **Phenylbutazone** | CYP2C9 |  | NA |  |  |  |  |  |  |  |
| **Sulfamethizole** | CYP2C9 |  | NA |  |  |  |  |  |  |  |
| **Suprofen** | CYP2C9 |  | CYP2C9 |  |  |  |  |  |  |  |
| **Tenoxicam** | CYP2C9 |  | CYP2C9 |  |  |  |  |  |  |  |
| **Trimethoprim** | CYP2C9 |  | CYP2C8 |  |  |  |  |  |  |  |
| **Zafirlukast** | CYP2C9 |  | CYP2C8 | CYP2C9 |  |  |  |  |  |  |

**Table S9:** Performance of SVM models developed on CDK and Vlife descriptors, models evaluate using fivefold cross-validated technique. Prediction is based on single label prediction.

| **Descriptor**  **Calculation** | **Parameter** | **Overall Accuracy%** | **Average Accuracy%** |
| --- | --- | --- | --- |
| CDK | d =1 , j = 2, c = 1 | 81.42 | 86.60 |
| Vlife | d = 0.005, j = 10, c = 0.05 | 80.58 | 84.50 |

**Table S10:** Percent of correctly predicted substrates (Accuracy) belongs to different CYP isoforms where only single isoform was predicted for each substrate/molecule

| **CYP Isoform** | **CDK descriptors** | **Vlife descriptors** |
| --- | --- | --- |
| CYP 1A2 | 87.16 | 85.65 |
| CYP2C9 | 88.99 | 91.39 |
| CYP2C19 | 90.37 | 89.95 |
| CYP2D6 | 84.86 | 82.78 |
| CYP3A4 | 81.65 | 72.73 |

**NOTE:** From the Table- S9 and Table-S10 we conclude that CDK calculated 26 best descriptors perform well as compare to Vlife calculated 23 selected descriptors, so in next step of study, for the prediction of multiple CYP isoforms we used CDK calculated 26 descriptors for SVM models development. List of 26 descriptors with their calculated values are available on the MetaPred server (http://crdd.osdd.net/raghava/metapred/) as an output of given molecule.

**Table S11**: Performance of SVM models developed for different CYP isoforms, all models evaluated using fivefold cross-validation technique.

| **CYP Isoform** | **Sensitivity%** | **Specificity%** | **Accuracy%** | **MCC** |
| --- | --- | --- | --- | --- |
| CYP3A4 | 72.41 | 75.66 | 75.23 | 0.35 |
| CYP2D6 | 80.00 | 81.31 | 81.19 | 0.41 |
| CYP1A2 | 64.71 | 68.16 | 67.89 | 0.19 |
| CYP2C9 | 73.91 | 76.74 | 76.15 | 0.44 |
| CYP2C19 | 83.96 | 80.36 | 82.11 | 0.64 |
| **Average** | **74.99** | **76.45** | **76.51** | **0.41** |
